# Forage-Poisoning—Ergotism

**Published:** 1901-07

**Authors:** Jacob Helmer

**Affiliations:** Scranton, Pa.


### FORAGE-POISONING—ERGOTISM.

By Jacob Helmer, D.V.S.,
SCRANTON, PA.

The two following interesting cases were given by Dr. Helmer in his report as chairman of the Committee on Sanitary Science and Police, at the annual meeting of the Pennsylvania Veterinary Medical Association on March 5, 1901.

### Forage-poisoning.

On Sunday, November 18, 1900, we received a hurry call from W. C. Adams, Chinchilla, Lackawanna County, Pa. Mr. Adams' herd consisted of thirty-eight head. We were called to see the fourth victim. Three cows had died during the past twelve days. The patient was a seven-year-old grade Jersey that lay stretched upon the floor. The symptoms appeared suddenly, and, as shown by the case, were as follows: Pulse frequent and weak, temperature 104°, and respirations increased. There was grating of the teeth. The eyeballs were sunken in their sockets and the cornea appeared lifeless. The urine was scanty. The bowels constipated and the secretion of milk had ceased. The victim was in a semi-comatose condition, alternating with attacks of delirium. At these periods the animal made ineffectual attempts to rise and struggle violently. The weakness of the posterior part of the body was so great that the cow could only raise herself up a little in front. There was more or less difficulty in swallowing.

About this time another case was discovered. A two-year-old heifer began to show the same initial symptoms. The pulse was frequent and weak, and the respirations hurried. This animal plunged and reared in her stanchion. The eyes were glassy and blind. There was profound twitching of the muscles of the eyes and of the body. The legs were braced and stiff, to prevent falling. The violent symptoms lasted but a few moments, and were followed by an almost complete cessation of phenomena. This case further on had vertigo and delirium by spells until recovery took place.

All the cases showed practically the same symptoms as the ones just described, differences depending upon the intensity of the attack and upon the parts affected. Two died in a comatose condition in from twenty-four to thirty-six hours. Case IV., the one first seen by us, died the following morning. During the next ten days three new cases appeared, all of which recovered.

An examination of the surroundings and of the water-supply for the herd gave negative results. That the malady was caused by the food seems very probable. An examination of the grains fed shed no light. But we found that, instead of hay, cut cornstalks and leaves of sweet corn were being used. The stalks and leaves had been only partly dried in the field, and were yet somewhat green when brought into the barn and passed through the cutting box. Cut into short ends, the stalks and leaves were thrown together in one place, and constituted a mass of several tons. It had lain about two months and was found to be badly fermented in spots or sections. In such places a bluish-green mould was very prolific. The outside of the pile seemed normal. The inside furnished the poison fungus which was held to be the cause of the disease. The cattle had been fed on the corn-stalk diet for two weeks previous to the appearance of any symptoms.

In order to halt the outbreak, its apparent cause, the fungus of the corn-fodder, was overcome by the interdiction of the use of any food upon which the mould was found. The treatment adopted consisted mainly in the employment of physic, stimulants, and, also, the administration of carbolic acid.

My brother, Dr. R. C. Helmer, assisted in the treatment of these cases, and I am indebted to him for a description of some of the symptoms.

### Ergotism.

On February 9, 1901, we were called to the farm of Mr. G. W. Collins, Hoadleys, Wayne County, Pa. My brother, Dr. Robert

C. Helmer, answered the call, and on his return reported the following interesting facts :

The trouble was with a small herd of cows. Fortunately there were only four head in the herd, of which three were affected with, to us, an unusual malady. The first case, a four-year-old common grade milk cow, had been already afflicted three weeks. The trouble began by lameness, showing itself first in one, later another, until all four legs were more or less diseased. The animal was dull and assumed, as a rule, the recumbent position. The first symptoms were heat, tenderness, and swelling in the affected extremities. This was followed a few days later by coldness, numbness, and loss of sensibility. This abnormal condition extended up to the knee-joint on the front legs and to the hocks on the posterior ones. In this case the skin was not broken, and showed no crack or slough.

Case II. had been affected two weeks, but only in the posterior extremities. Just above the cold and insensible skin swelling and heat with pain on pressure were manifest.

Case III. had been affected four days and in one limb only up to the ankles.

The temperature as observed in these cases was normal. All the functions were normal ; mucous membrane showed slight anæmia. The diagnosis made was that of chronic ergotism caused by feeding on ergotized grasses. A species of red-top, on which the cattle were being fed, contained ergot in considerable quantities. The cases were put on local and internal treatment, and when seen last, on February 12th, all had improved and showed good prospects of making a complete recovery.

On February 22d last we found another outbreak on the farm of Daniel Swingle, of Lake Ariel, Wayne County, Pa. Three cows were already dead, two of them had lost their front hoofs and had to be destroyed, the third showed disordered digestion, nervous symptoms, and gangrenous ergotism of the extremities. This case lived but a few days. At the time of our visit two cases were found, one of which showed a complete slough of the soft tissues below the hock, exposing the bone, which also took part in the gangrenous process. Another case showed a complete slough around the ankle-joint of the right hind leg. It hung loose and could easily have been pulled off. Both of these victims were ordered destroyed. The three remaining members of the herd were not affected. An examination of the hay, which was mixed timothy and clover, showed no suspicious characters. At this point Mr. Swingle explained that he had during the past few weeks been feeding red-top, but that his stock of it had become exhausted. A forkful was, however, found in the hay-mow, and showed quite an abundance of ergot. They began feeding ergotized red-top on December 12, 1900, and it was not exhausted until January 15, 1901. The first three cases were lost during the latter part of December, and the last two about December 25th and died on January 15th. Since the diet was changed no new cases have developed.

A small sample of the red-top was immediately sent to Dr. Pearson, the State Veterinarian, whose reply was as follows:

HARRISBURG, PA., February 26, 1901.

DR. JACOB HELMER, 311 Spruce Street, Scranton, Pa,

DEAR DOCTOR: In reply to your recent favor I beg to advise you that I have received from Dr. J. W. Harshberger the following report:

"I have examined the sample of red-top (agrostis alba), and find that in place of many of the grains there is a small growth of a species of ergot (claviceps microcephala, Walls), which would be sufficient to account for the character of the disease you mention. I shall be much interested to hear from you again as to the results of your treatment. It appears to me the evidence of ergotism is very strong.

"Very truly yours,

"LEONARD PEARSON."

---

A TEN THOUSAND DOLLAR ENDOWMENT FOR THE FLOWER VETERINARY LIBRARY.—The Roswell P. Flower Veterinary Library, founded by a gift of $5000 by the late Governor for the use of the New York State Veterinary College, has just been endowed by Mrs. Flower with $10,000.

The library now contains 1754 bound volumes, mostly recent and up-to-date in their respective subjects, and forming a collection of the very highest value to the student and investigator in comparative medicine and allied subjects. Taken in connection with the large accumulation of books in the same fields in the University Library, this makes the New York State Veterinary College an unexcelled field for study and research, and it is confidently believed that the constant additions of the best modern publications will keep the library at all times up to date as a unique centre of contemporary knowledge and achievement.

